# Single-Atom Engineering in Room-Temperature Sodium–Sulfur Batteries

**DOI:** 10.1021/accountsmr.5c00172

**Published:** 2025-09-02

**Authors:** Bindu Kalleshappa, Martin Pumera

**Affiliations:** † Future Energy and Innovation Laboratory, Central European Institute of Technology, 48274 Brno University of Technology, Purkyňova 123, 61200 Brno, Czech Republic; b Advanced Nanorobots & Multiscale Robotics Laboratory, Faculty of Electrical Engineering and Computer Science, VSB - Technical University of Ostrava, 17. listopadu 2172/15, 70800 Ostrava, Czech Republic

## 1 Introduction

Single-atom engineering (SAE) is a novel technique of modifying a molecule with a single active center to enhance its functionality. SAE has emerged as a transformative advancement in the field of catalysis, characterized by the dispersion of individual metal atoms on support materials such as metal oxides, carbon-based substrates, or other stable matrices.^1,2^ A schematic of single-atom engineered (SAE) materials on different matrices is shown in Figure 1. In SAE, each metal atom is kept isolated and accessible for catalytic reactions unlike in nanoparticle or bulk metal clusters, thereby maximizing the utilization of active sites. The atomic-level dispersion enhances the catalytic efficiency and controls the precise electronic properties of the catalysts, which leads to superior performance in various chemical transformations and energy-related applications.^1^

**Figure 1:**
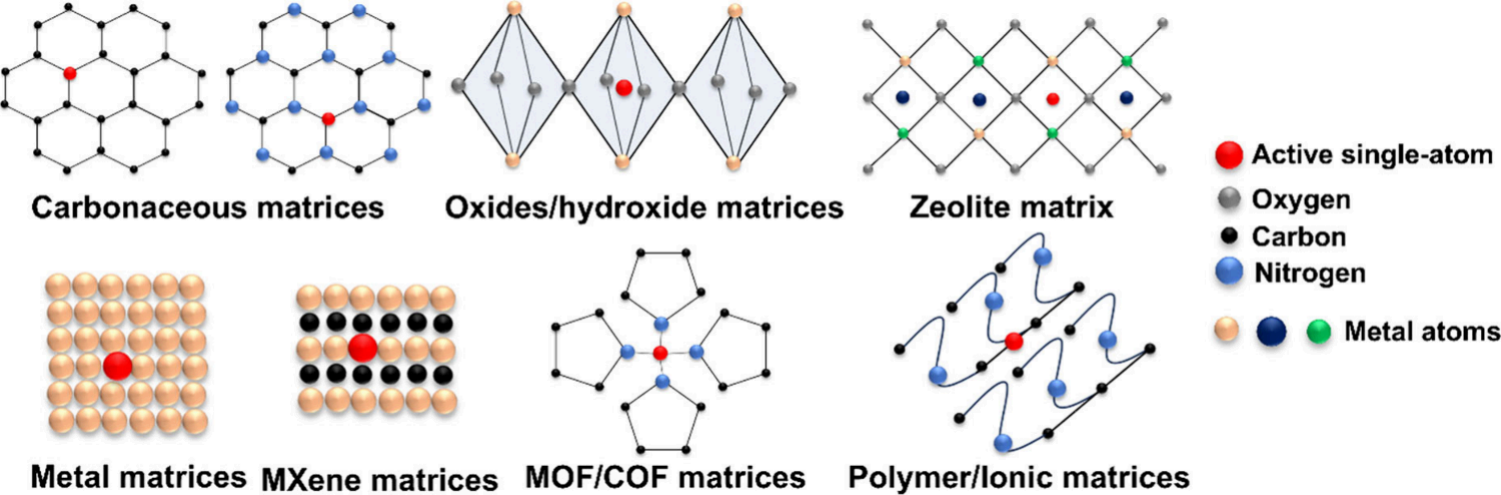
Schematic representation of different kinds of single-atom engineered materials based on their matrices.

As the size of metal species decreases, the specific activity of the species increases due to the surface free energy of the metal species increasing as the bulk material turns into nanoparticles, sub-nanoparticles, and single atoms.^3^ Single-atom metal sites are highly chemically interactive, attributed to the size effects of the metal nanocatalyst. In SAE materials, the presence of highly active valence electrons, pronounced quantum confinement effects, and the discrete electronic states of isolated metal atoms collectively result in a maximized surface free energy of the metal species. This elevated surface energy enhances chemical interactions with the support, thereby endowing in SAE materials with their unique chemical properties.^4^

SAE is transforming fields from catalysis and energy storage to quantum information, nanoelectronics, sensing, and biomedicine by allowing us to place, probe, and program individual atoms as functional units. Its contribution to energy storage devices like lithium-sulfur (Li–S) and sodium-sulfur (Na–S) helps to overcome the drawbacks of these battery systems. This Viewpoint explores potential ways of designing and engineering single-atom centers to mitigate the challenges of Na–S batteries and to enhance their performance.

## 2 Room-Temperature Na–S Batteries

Although lithium-based energy-storage systems lead in high-performance metrics, they cannot serve the demand of energy storage devices in the long term due to the limited geographical concentration of lithium sources.^5^ Limited distribution and increasing consumption are leading to the depletion of lithium resources and an increase in price.^6^ Additionally, lithium batteries depend on expensive raw materials and have insufficient energy density for large-scale grid applications, leading to the development of alternative energy storage systems.^7^ As a result, sodium-based technologies are a better choice due to their lower cost, abundant feedstocks, and sustainability advantages. Room-temperature Na–S batteries have emerged as a promising technology, boasting high theoretical capacities for both sodium (1166 mAh g^–1^) and sulfur (1672 mAh g^–1^) and thus possessing a high theoretical energy density of 1274 Wh k g^–1^. Moreover, sodium can be easily extracted from seawater, while sulfur, the 16th most abundant element, can be found in volcanic deposits. Room-temperature Na–S cells offer an environmentally friendly pathway for large-scale applications, unlike lithium-ion batteries.^8^ Room-temperature Na–S batteries benefit from a higher theoretical energy density (1274 Wh kg^–1^) due to the final discharge product being Na_2_S.^9^

### 2.1 Challenges of room-temperature Na–S batteries

Despite their advantages, room-temperature Na–S batteries face challenges related to low Coulombic efficiency, self-discharge, and capacity fade over prolonged cycling. These problems primarily arise from the dissolution of polysulfides in the electrolyte and the shuttle effect, where soluble polysulfides (PSs) diffuse to the anode, causing side reactions and the loss of active material. Additionally, the transformation from S_8_ to Na_2_S involves large volumetric changes, compromising the structural integrity of the electrode.^10,11^

The challenges in room-temperature Na–S batteries can be overcome by different approaches, for example, (i) designing a suitable current collector with high mechanical and chemical strength and anticorrosive nature, (ii) designing a suitable electrolyte with fast sulfur conversion and low side reactions, (iii) designing a suitable separator for faster ionic flow with high mechanical strength, and (iv) adding electrocatalysts to improve sulfide conversion kinetics.^11,12^

Various strategies have been proposed to mitigate polysulfide dissolution and the shuttle effect, including advanced electrode materials to confine sulfur and polysulfides, electrolyte optimization to stabilize the SEI, and modifications to cell design (e.g., improved separators) in different metal–sulfur systems. Progress in nanomaterials, computational modeling, and state-of-the-art characterization techniques continues to drive innovation in this area, aiming to achieve a higher energy density.

## 3 Single-Atom Engineering (SAE) to Overcome Challenges

Specifically, in Na–S batteries, SAE has significant potential to address inherent challenges such as the shuttle effect and the low kinetics of sulfur conversion. Figure 2 shows a schematic representation of the contribution of single-atom catalysts in overcoming challenges of room-temperature sodium–sulfur (Na–S) batteries. Current research in single-atom engineering focuses on designing single-atom catalysts to enhance the catalytic conversion of sodium polysulfides, thereby improving reaction kinetics and reducing the formation of unwanted byproducts. Higher efficiency and reliable battery performance can be achieved by improving the electrochemical kinetics and structural integrity of battery components through precisely tailored active sites of single-atom centers and optimized catalytic processes involved in sulfur redox reactions.

**Figure 2:**
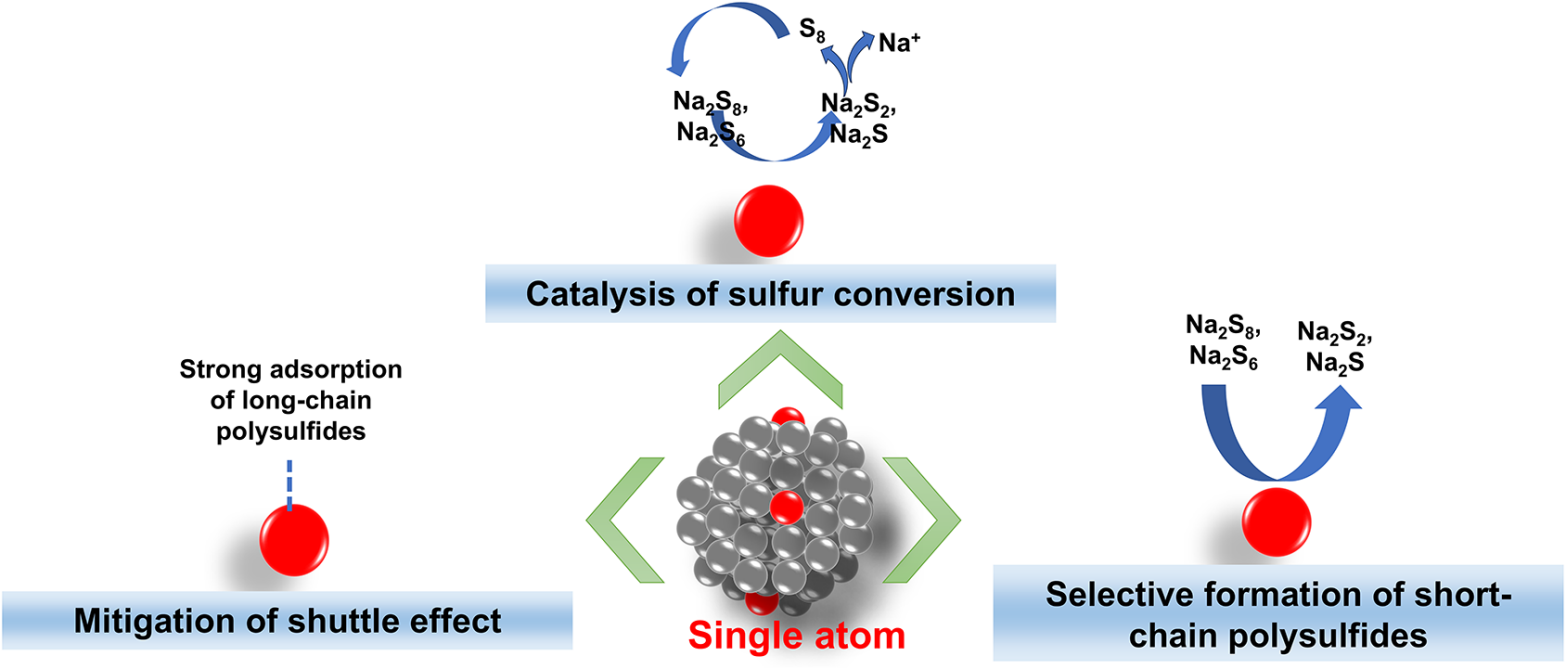
Schematic representation of contribution of single-atom in overcoming challenges of room-temperature sodium–sulfur (Na–S) batteries.

SAE can enhance the sulfur conversion of Na–S cells by tailoring single-atom centers and the environments of isolated metal centers on matrices. For example, enhanced electron redistribution around metal centers and optimized orbital interaction with matrices in symmetric planar configurations lead to faster sulfur redox reactions. Additionally, the sodiation process can be promoted by designing single-atom centers with antibonding orbitals, which have a higher tendency to accept electrons from polysulfide chains.^13,14^

The challenges in Na–S batteries are interconnected. If an SAC can adsorb the long-chain polysulfide intermediates and convert them into short-chain polysulfides, it is efficient in mitigating the shuttle effect, catalyzing sulfur conversion and the selective formation of short-chain polysulfides. Currently, most researchers employ the strategy of modifying the cathode with SAE to enhance the performance of Na–S batteries. Different metal-center single atoms actively accelerate sulfur conversion kinetics by improving electron transfer in sulfur cathodes. The distinct local structures formed by various metal atoms significantly influence their electronic properties, thereby modifying the adsorption energies and reaction barriers of sulfur and sodium sulfides on single-atom sites. Interestingly, different transition metal single-atom centers are effective in different single-atom catalyst environments.^13–16^ The metal center coordination bonds strongly confine sulfur, which in turn enhances the electrical conductivity and stability of the sulfur cathode. They are selective to the conversion of long-chain polysulfides to short-chain polysulfide without the formation of intermediates, which results in high reversibility of Na–S batteries.

Single-atom centers effectively anchor sodium polysulfide (NaPS) intermediates with lower reaction energy and mitigate the detrimental shuttle effect in Na–S systems. Single-metal-atom centers weaken the sulfur–sulfur bonds in the S_8_ ring, facilitating the catalysis of short-chain sulfur molecule formation. They also disintegrate long-chain polysulfides into short-chain polysulfides based on polar–polar interactions.

The electronic environment of both single-atom centers and matrices plays a major role in adsorbing intermediate polysulfide species and conversion kinetics. Optimized orbital interactions promote stronger and stable adsorption of polysulfide intermediates, accelerating their redox reactions, and thereby improving the kinetics and overall performance of Na–S batteries. In asymmetrical systems, transition metal single-atom centers form strong d–p orbital hybridization with sulfur atoms and accommodate p-orbital electrons of NaPSs due to their empty d-orbitals, leading to strong adsorption of NaPSs on metal centers. Similarly, the alignment of s-orbitals of NaPSs with p-orbitals of single-atom centers facilitates efficient charge transfer, lowering energy barriers associated with sulfur species conversion during redox cycles.^16–18^ Different single-atom design and engineering strategies should be adopted to address targeted challenges in Na–S battery systems.

### 3.1 Theoretical/Computational Tools to Support SAE

Density functional theory (DFT) calculations are major theoretical tools used to study the behavior of materials in different chemical environments. Reaction pathways, product formations, and other properties can be predicted using these calculations. The results provide energy levels, energy densities, or energy differences in different chemical environments. This predictive capability can be used to design and engineer single-atom metal centers for Na–S batteries to achieve elevated performance. For instance, first-principals calculations revealed that targeted engineering of metal centers in MXene hosts significantly boosts anchoring and catalytic activity, ensuring stronger adsorption of sodium polysulfides and efficient electron transfer at electrode surfaces. Introducing transition metal single atoms into the MXene (Ti_2_CS_2_) matrix markedly increases chemical interactions during the adsorption of S_8_/Na_2_S_*x*_ on catalysts, considerably enhancing the catalytic performance of pristine Ti_2_CS_2_ toward sulfur reduction reactions and thereby benefiting the rate capability and cycling stability of Na–S batteries.^19^ Different 2D materials to be studied as matrices to add single-atom which can lead to suppress the challenges of Na–S systems.

By systematically screening various single-atom configurations using first-principles calculations, transition metal single-atoms reduce the energy barriers for Na_2_S absorption and increase electrocatalytic decomposition rates, drastically boosting Na–S battery performance.^20^ Intrinsic mechanism analyses using DFT calculations revealed that d-orbital interactions of vanadium with p-orbitals of sulfur weaken Na–S bonds, inhibiting the shuttle effect and promoting Na_2_S dissociation.^21,22^

### 3.2 In Situ (Operando) Techniques to Support SAE

To validate computational and experimental predictions, advanced in situ/operando characterization tools such as X-ray absorption spectroscopy (XAS), high-resolution transmission electron microscopy (HR-TEM), and scanning transmission electron microscopy (STEM) are essential. These techniques provide real-time visualization of evolving electrochemical and structural processes, crucial for evaluating transient phenomena caused by the operating environment of Na–S batteries, including dynamic changes in chemical composition, intermediate species formation, and electrode–electrolyte interactions that dictate battery performance.

In situ XAS in Na–S batteries reveals changes in oxidation states of single metal atoms during cycling, helping to identify degradation pathways and understand in SAE materials catalytic mechanisms. Similarly, operando Raman spectroscopy monitors the formation and consumption of polysulfide species during cycling. In situ X-ray diffraction (XRD) tracks phase changes in the sulfur cathode during polysulfide formation and decomposition. Additionally, in situ TEM visualizes isolated or agglomerated single atoms and structural changes during battery operation. In situ/operando techniques facilitate real-time observation of SACs under operational conditions, evaluating catalyst stability, reactivity, mechanism elucidation, degradation pathways, and correlative analysis in Na–S batteries. SAE design principles for maximum performance in Na–S batteries can be fine-tuned by correlating in situ data with theoretical insights.^13–17,23,24^

## 4 Concluding Remarks and Outlook

Single-atom engineering redefines the reaction landscape of room-temperature sodium–sulfur batteries by offering a catalysis-centric solution to long-standing issues of polysulfide dissolution and sluggish kinetics. High sulfur utilization, higher rate capability, and prolonged cycle life of Na–S batteries can be achieved by tuning active sites through single-atom engineering. Single-atom centers excel in polysulfide anchoring and conversion, shuttle effect mitigation, and stabilization of the electrode–electrolyte interface over extended cycling.

Looking forward, SAE of electrolytes with single-atom active centers could further mitigate dendrite formation and stabilize anode–cathode interfaces. Eventually, incorporating the advantages of single-atom engineering into advanced separator technologies, optimized electrolytes, and robust electrode designs could bring room-temperature Na–S batteries closer to widespread deployment for large-scale, cost-effective energy storage. Although SAE exhibits considerable promise for boosting Na–S battery performance, several challenges and knowledge gaps remain.

Long-term stability remains an open challenge for many single-atom engineered cathodes. Achieving high areal capacities, extended cycle life, and robust shelf life of Na–S batteries must be addressed with advanced single-atom engineering techniques. Under repeated cycling, single-atom sites may migrate, aggregate, or undergo chemical changes, especially in the presence of corrosive polysulfides and volume fluctuations in electrodes, thereby diminishing the initial high catalytic activity. This degradation mechanism can be worsened by repeated expansion and contraction of sulfur cathodes and side reactions with dissolved polysulfides. Maintaining the structural integrity of single-atom catalytic centers during repeated sodium plating/stripping cycles is crucial for eventual commercial adoption. Further research on reinforcing metal–support interactions and designing robust electrolyte systems is needed to ensure stable performance over thousands of cycles. Additionally, investigations should extend beyond coin-cell demonstrations to pouch or cylindrical cells under realistic operating conditions such as temperature, pressure, and cycling rates.

In summary, single-atom engineering stands out as one of the most promising solutions for elevating room-temperature Na–S battery technology to meet industrial demands. Integrating atomic-scale design, electronic structure engineering, and system-level optimization can pave the way for a new generation of high-performance, cost-effective, and durable sodium–sulfur batteries. Continued interdisciplinary collaboration spanning theoretical modeling, advanced materials synthesis, and operando characterization will be key to translating these scientific breakthroughs into commercially feasible energy storage solutions.
